# Main topics in assisted reproductive market: A scoping review

**DOI:** 10.1371/journal.pone.0284099

**Published:** 2023-08-01

**Authors:** Janaina Ferreira Aderaldo, Beatriz Helena Dantas Rodrigues de Albuquerque, Maryana Thalyta Ferreira Câmara de Oliveira, Mychelle de Medeiros Garcia Torres, Daniel Carlos Ferreira Lanza

**Affiliations:** 1 Applied Molecular Biology Lab (LAPLIC), Biochemistry Department, Federal University of Rio Grande do Norte (UFRN), Natal, Rio Grande do Norte, Brazil; 2 Januário Cicco´s University Hospital (MEJC), Federal University of Rio Grande do Norte (UFRN), Natal, Rio Grande do Norte, Brazil; China Agricultural University, CHINA

## Abstract

**Background:**

Infertility affects around 12% of couples, and this proportion has been gradually increasing. In this context, the global assisted reproductive technologies (ART) market shows significant expansion, hovering around USD 26 billion in 2019 and is expected to reach USD 45 billion by 2025.

**Objectives:**

We realized a scoping review of the ART market from academic publications, market reports, and specialized media news, to identify the main terms and characterize them into the main topics in the area.

**Design:**

We apply an LDA topic modeling process to identify the main terms, and clustered them into semantic synonymous topics. We extracted the patterns and information to these topics and purposed a factor/consequence correlation to them.

**Results:**

We found 2,232 academic papers and selected 632 to include in the automatic term detection. We also included 34 market reports and seven notices produced by specialized enterprises. Were identified 121 most relevant cited terms covering 7,806 citations. These terms were manually aggregated into 10 topics based on semantic similarity: *neutral terms* (37.2%), *economic aspects* (17.6%), *in vitro fertilization* (*IVF) commodities & cross-border reproductive care (CBRC)* (10.6%), *geographic distribution* (9.5%), *social aspects* (7%), *regulation* (6%), *trends & concerns* (3.9%), *accessibility* (3.4%), *internet influence* (2.9%), and *fertility preservation for non-medical reasons* (2%).

**Discussion:**

The analysis indicates a market with expressive complexity. Most terms were associated with more than one topic, indicating the synergism of this market’s behavior. Only seven terms related to economic aspects, surrogacy and donation represent around 50% of the citations. Except for the topic formed by generic terms, the topic of the economic aspects was the most represented, reflecting macro perspectives such as a-la-carte standard of treatments, many clinics operating on a small/medium scale, and the recent formation of conglomerates. The IVF commodities & CBRC topic brings an overview of gametes pricing and transnational surrogacy, and its regulation. The topic of geographic distribution indicates that that the Asia-Pacific (APAC) market has the most significant growth potential in all fields. Despite the increase in supply and demand for infertility treatments and technological advances in recent decades, the success rate of IVF cycles remains at around 30%. Terms referring to research and development or technical improvement were not identified in a significant way in this review.

**Conclusions:**

The formation of topics by semantic similarity proved to be an initial path for the elaboration of in-depth studies on the dynamics between several factors, for this, we present the panel classifying main terms into factors (demand, pent-up demand, or distributive) or ART market consequences. Through this approach, it was possible to observe that most of the works addresses economic aspects, regulation and geographic aspects and that topics related to research and improvement have not been addressed. In this way, we highlight the need to deepen the analysis of market elements that may be related to increased efficiency of IVF in the technical field.

## Introduction

### Rationale

Infertility is currently defined as a failure to achieve a clinical pregnancy after at least one year of regular attempts [1], which was later updated to include physiological or psychological conditions incompatible with natural meeting of gametes [2]. It affects between 8% and 12% of couples globally [3], and this proportion is gradually increasing [1, 4] because of multiple causes, such as the modern lifestyle, diseases, and the postponement of parenthood [5–7].

In that context, it is estimated that half of the infertile couples never seek fertility [8], and the investigation of the reasons reveals a complex product of the national public and private health policies and economic, political, and cultural factors [9–11].

However, the global assisted reproductive technology (ART) market expanded in clinic numbers and procedures [9], The ART market services were around USD 26 billion in 2019 [12] and are expected to reach USD 45 billion by 2025 [5]. Between 1997 and 2016, ART treatments have increased more than five-fold in Europe, 4.6-fold in North America, and three-fold in Australia and New Zealand [13, 14].

The distribution of this billionaire market is heterogeneous due to complex clustering factors like unequal regulatory restrictions, local procedures practices, and socio-cultural differences associated with disposable income [7, 15–18]. Despite the volume of information about several factors that compose this market, there is no structured analysis of these factors in clusters.

For this reason, we produce this scoping review for the identification of the main terms and topics cited in ART market texts. This is an appropriate tool for examining emerging evidence that has not been comprehensively reviewed or of a complex and heterogeneous nature, mapping the available evidence for clarifying definitions and conceptual boundaries [19, 20].

### Objectives

The following question guided this review: What are the aspects that compose the global ART market?

## Methods

To answer the question that guided this review, we choose the scoping review approach with Latent Dirichlet Allocation (LDA) topic modeling as the method to identify this evidence.

### Protocol

**Fig 1:**
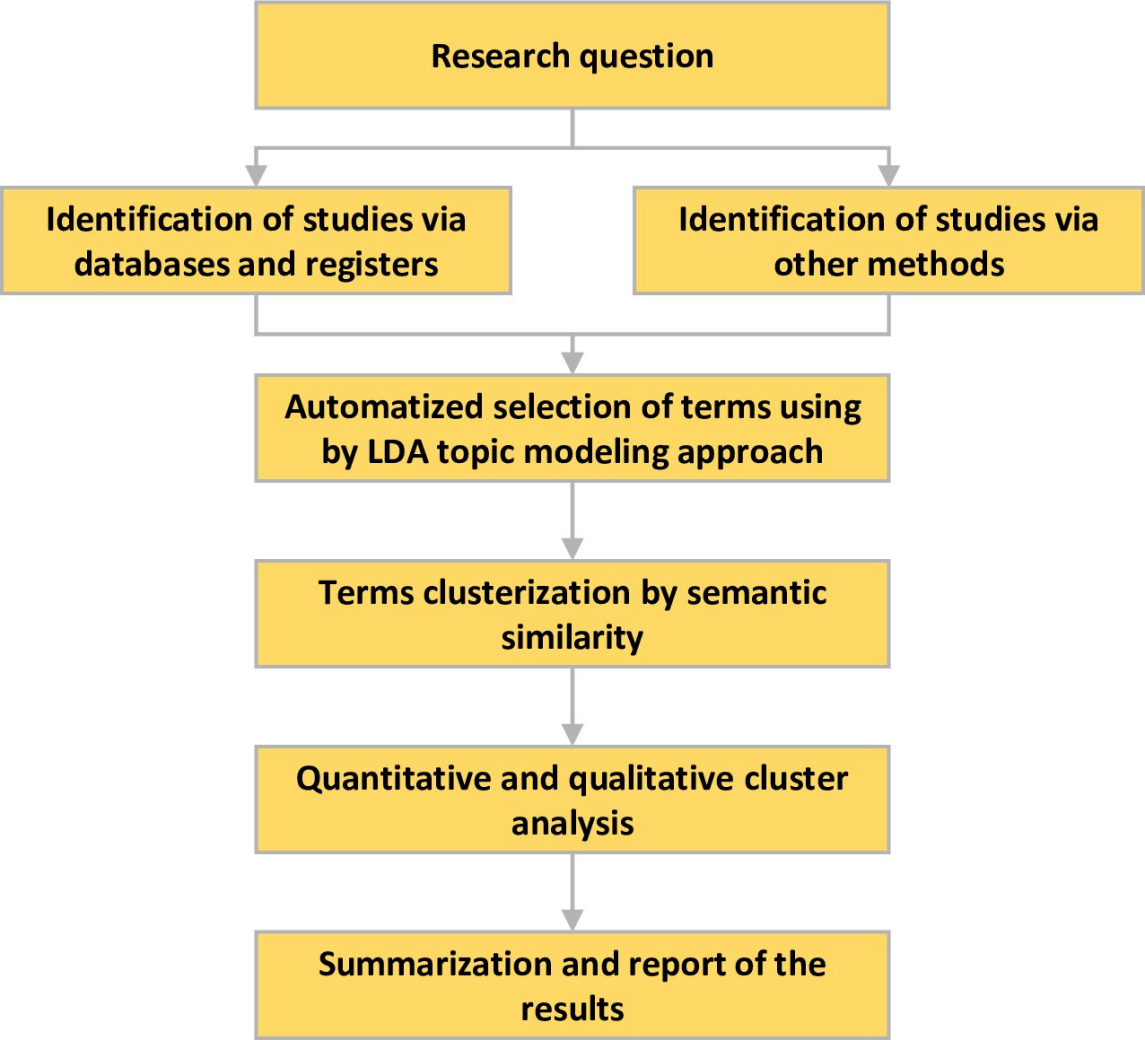
Study design for scoping review research (Prisma-ScR) with a topic modeling approach. This design was adapted from Page et al. (2020) and purposes to identify the studies in databases and other methods through the query elaborated from the research question. After selecting studies by eligibility criteria, the titles, keywords, and abstracts were subjected to detecting terms using the topic modeling approach [23]. Terms were aggregated by semantic similarity into clusters (topics), summarized, and presented.

We performed a scoping review based on guidelines proposed by the Joanna Briggs Institute (JBI) Scoping Review Methodology Group [21]. The methodology was adapted from Tricco *et al*. (2017) [22]: a) elaboration of the research question; b) identification of relevant studies; c) selection of relevant terms by LDA topic modeling using an automatic tool and aggregation of them by iterative team approach for studying a selection and data extraction [23, 24]; d) chart production from the data incorporating quantitative and qualitative thematic analysis; e) summarization and report of the results (Fig 1).

### Eligibility criteria

We included the following peer-reviewed and gray literature in this review: a) academic publications; b) market reports about the ART market produced by specialized research companies; and c) selected referenced media news.

About peer-reviewed publications, the following bibliographic databases were screened from 2010 to 2022: PUBMED, MEDLINE, EMBASE and Google Scholar. We defined the query (((assisted reproductive market) OR (infertility market)) OR (fertility market)) AND (("01/01/2010"[Date—Publication]: "2022"[Date—Publication]))).

For gray literature, market reports, and media news, we searched Google using the terms ‘assisted reproductive market’, which focused on referenced economic agency websites, and specialized media websites, excluding blogs and clinic websites.

### Search and selection of sources of evidence

Two independent reviewers selected pertinent literature through abstracts and titles using the Sysrev software [25]. Disagreements were resolved by consensus. After this selection, the selected studies were submitted to LDA topic modeling from the content of abstracts, title, and keywords using keywords Knime software [24], which identifies repetitive word patterns across a corpus of documents [26].

### Synthesis of results

The terms detected by topic modeling were clustered by synonymous and semantic similarity into topic groups. We evaluate the content of these topics and present them in a quantitative approach by ranking the number of term citations in each topic about the recent ART, and a qualitative approach through an analytic mini review.

### Methodological quality appraisal

We did not appraise methodological quality or risk of bias of the included articles, consistent with guidance on scoping review conduct [20]. We draw the Preferred Reporting Items for Systematic reviews and Meta-Analyses extension for Scoping Reviews (PRISMA-ScR) checklist [27] in S1 Table.

## Results

### Selection of sources of evidence

**Fig 2:**
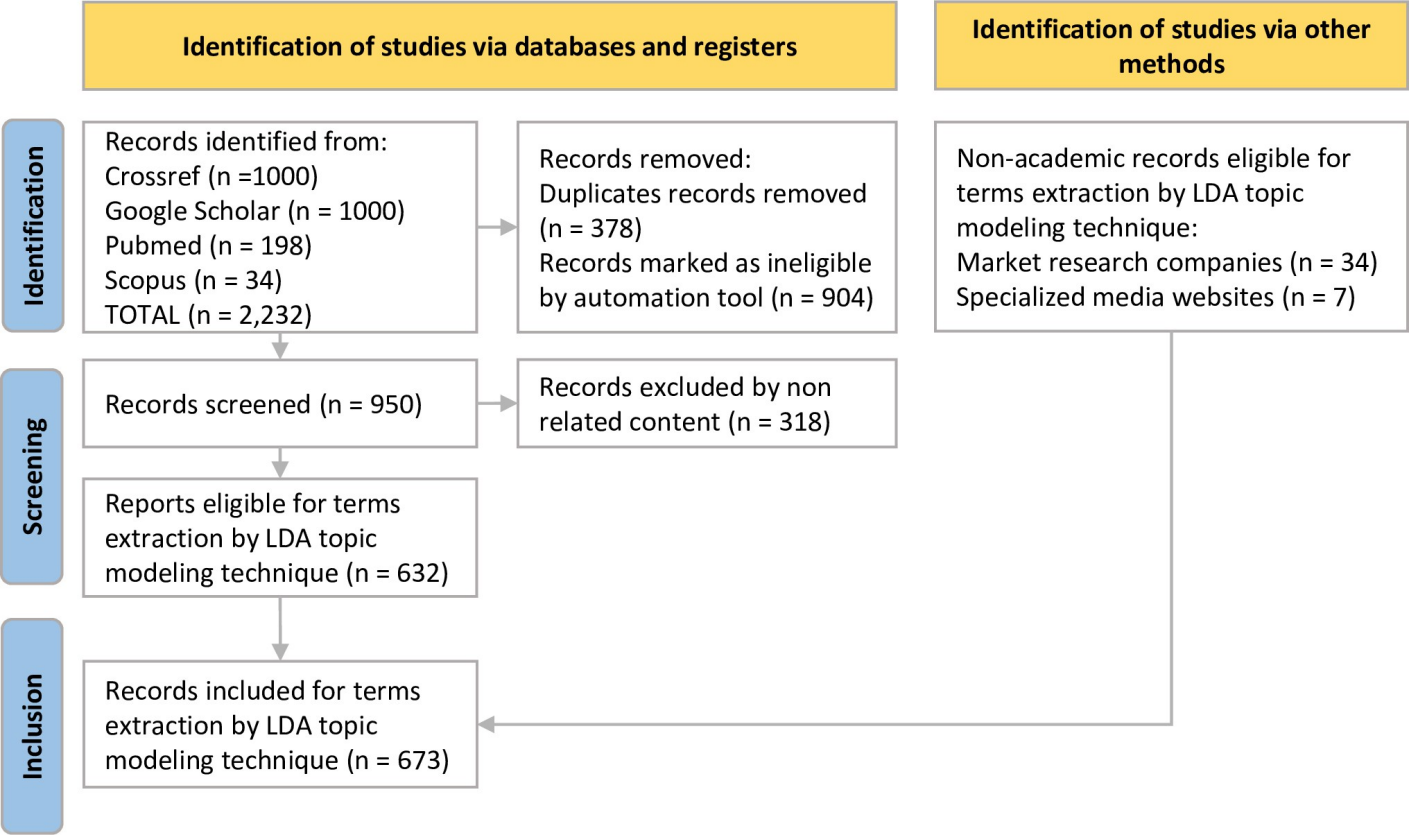
Flow diagram for scoping review research (Prisma-ScR). We identified 2,232 academic publications in initial search. After duplicates, and ineligible exclusions (n = 1,600), we included 41 gray literature records. In total, were selected 673 abstracts, titles, and keywords for topic modeling screening step.

We found 2,232 academic papers and selected 632 that were eligible. We also included 34 market reports and seven notices produced by specialized enterprises. In these 673 records, were identified 121 most relevant cited terms covering 7,806 citations (Fig 2). The academic evidence source represents 93.9% of the total, whose proportion remained approximate in the abundance of citations for each term.

### Synthesis of results

**Fig 3:**
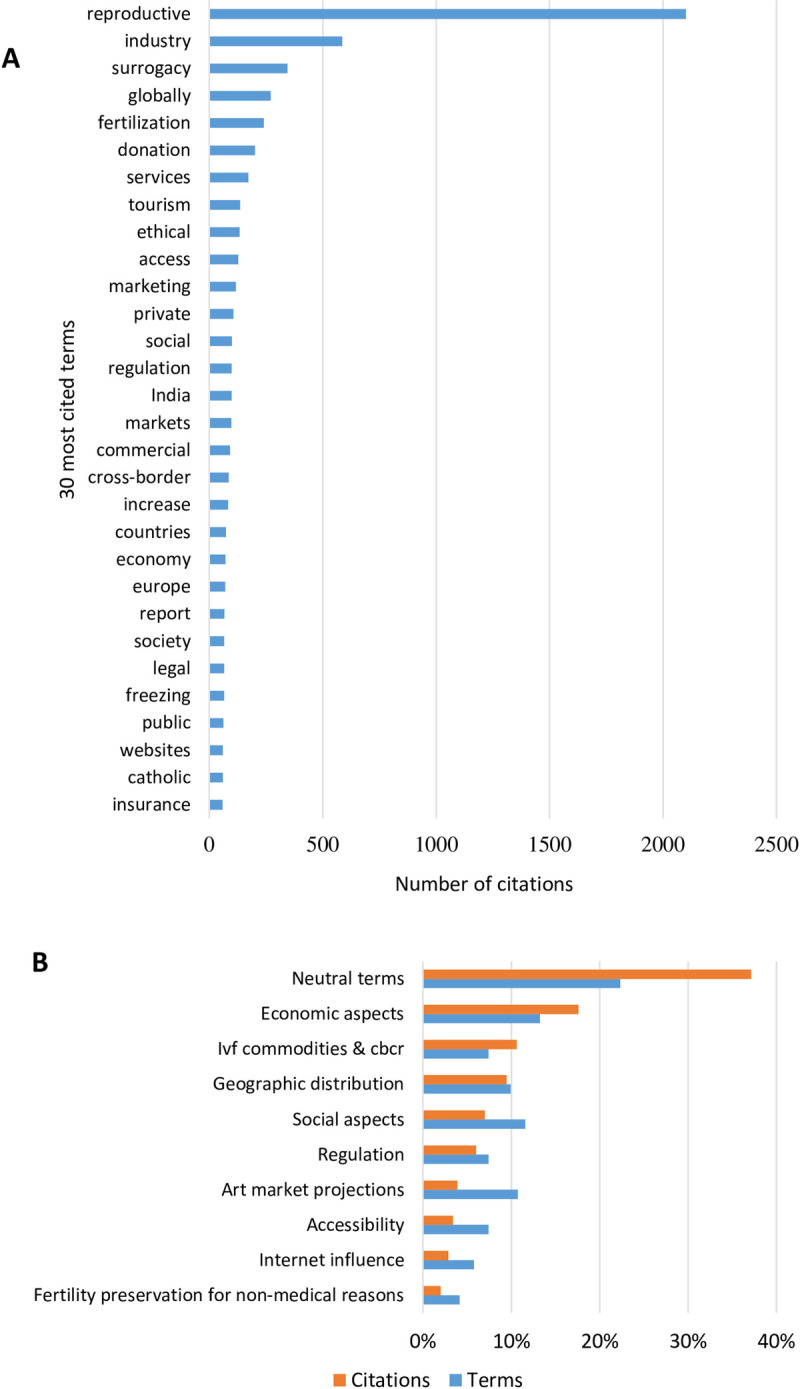
Detected terms and clustering. A) 30 most cited terms detected by the topic modeling automation tool and each number of citations on the database. 26.9% of citations correspond to the neutral term *reproductive*. This was overestimated for being present in all titles and keywords and repeated in abstracts. Disregarding this term, we identified specific terms such as *industry* (586 citations) and *surrogacy* (345 citations); B) 10 Clusters of 121 detected terms aggregated by semantic similarity. We chose to organize the clusters based on the total number of citations (orange bars). The number of topics in each cluster is available in the blue bars.

The 121 identified terms were cited 7,806 times in 673 texts used for the terms detection approach (Fig 3A and S2 Table). The ratios were 0.18 for terms/number of texts and 11.6 for the number of citations/number of texts. We manually aggregated by team consensus these 121 terms into 10 clusters by semantic similarity (Fig 3B and Table 1). As an example, the terms `ethical`and `social`were clustered into the topic of the social aspects.

**Table 1:** Topics clusters of the detected terms.

TOPICS	N° of citations	N° of terms	CLUSTERED TERMS
NEUTRAL TERMS	2900	27	Reproductive, gestational, conceive, patient, effects, genetic, reduced, decline, controls, pregnant, created, middle, conception, quality, parental, experience, improvements, population, delivery, counselling, estimated, methods, fertilization, abortion, adoption, increase, report.
ECONOMIC ASPECTS	1375	16	Marketing, markets, industry, services, economy, commercial, management, recession, business, Insurance, coverage, employment, providers, financial, benefits, costs.
COMPENSATION FOR REPRODUCTIVE SERVICES	829	9	Surrogacy, donation, banking, commodification, banks, payment, Tourism, cross-border, facilitators.
GEOGRAPHIC DISTRIBUTION	739	12	Globally, India, countries, Europe, transnational, travel, China, federal, Israel, Spain, worldwide, latin.
SOCIAL ASPECTS	546	14	Ethical, social, society, catholic, gender, queer, lesbian, diversity, concerns, moral, religion, feminist, religious, stress.
REGULATION	469	9	Regulation, legal, policy, unregulated, guidelines, private, public, decision-making, official.
ART MARKET PROJECTIONS	304	13	Constraints, opportunities, demand, trends, potential, concern, perspectives, exploitation, perceptions, considerations, comparison, implications, issues.
ACCESSIBILITY	264	9	justice, inequalities, choice, acceptability, disparities, conditions, access, stratified, equity affiliated.
INTERNET INFLUENCE	224	7	Websites, media, influence, internet, online, WeChat, information.
FERTILITY PRESERVATION FOR NON-MEDICAL REASONS	156	5	Preservation, freezing, clock, contraceptive, carrier.
10 clusters	7806 citations	121 terms	

The Table 1 brings the compilation of terms aggregated by semantic similarity, that is, direct synonyms or referring to the same theme or meaning. We call the assisted reproduction market topic clusters.

The *neutral terms* topic corresponds to 37.2% of citations (27 terms; 2,900 citations). Despite this topic presenting the majority cluster, these terms were disregarded for the content analysis because they returned practically the entire set of records used.

The *economic aspects* topic corresponds to 17.6% of citations (16 terms; 1,375 citations) and refers to the econometric analysis of the market. We identified two subgroups, which following: a) a group of nine generic terms (1,178 citations) that returned unspecified records, and b) a group of seven terms (197 citations) representing specific economic terms such as insurance and coverage.

The *compensation for reproductive services* topic corresponds to 10.6% of citations (9 terms; 829 citations) and refers to records about the pricing of reproductive services and their ramifications. We identified two subgroups: a) commercial surrogacy (4 terms; 574 citations), and b) gametes pricing (5 terms; 255 citations).

The *geographic distribution* topic corresponds to 9.5% of citations (12 terms; 739 citations) and refers to the global distribution of the market in terms of size, demand, characteristics, and profitability.

The *social aspects* topic corresponds to 7% of citations (14 terms; 546 citations). In this group, the terms can be clustered into five subgroups, which follows: a) ethical/moral discussions (4 terms, 314 citations); b) religious issues (3 terms, 84 citations); c) gender issues (3 terms, 82 citations); d) sexual preferences (2 terms, 36 citations); and e) stress that was considered an independent term (10 citations).

The *regulation* topic corresponds to 6% of citations (9 terms; 469 citations) and refers to legal aspects of the ART market. It includes the assessment of the impact of regulation (laws and guidelines) on market behavior and the assessment of the impact of transnational practices on national regulation.

The *ART market projections* topic corresponds to 3.9% of citations (13 terms; 304 citations) and refers to prognostic analyzes. The group can be divided into three subgroups: positive trends (5 terms; 115 citations), b) concerns (5 terms; 152 citations), and c) neutral comparisons (3 terms; 47 citations).

The *accessibility* topic corresponds to 3.4% of citations (9 terms; 264 citations) and refers to terms that returned a set of texts to evaluate the social impacts related to the ART market.

The *internet influence* topic corresponds to 2.9% of citations (7 terms; 224 citations) and refers to terms that returned a set of texts related to the evaluation of the internet in the ART market.

The *fertility preservation for non-medical reasons* topic corresponds to 2% of citations (5 terms; 156 citations) and refers to terms that returned a set of texts related to this specific theme.

## Discussion

### Critical appraisal within sources of evidence

Although resulting from the same search terms, we have found two essential differences in the content of academic publications and market studies. Academic publications focus, in general, on only one factor as a central objective and make an in-depth analysis. In contrast, market reports focus on economic projections and, more often, present the factors in a superficial and aggregated way as an *increase/decrease* ART market factor.

The specialized media news provided relevant information on the formation of conglomerates and open market companies. Considering that 93.9% of used literature was composed of peer-reviewed publications, the gray literature can provide a complementary perspective to peer-review information [28].

### Summary of evidence

The ratio of 0.18 between the number of terms/number of texts and 11.6 between the number of citations/number of texts inform us that the texts generally deal with specific themes, whose citations are reinforced throughout the texts. Of the 121 terms detected, 16 were identified as variations of the same lexical root, and the others were identified as synonyms. We checked the accuracy of the automated tool by comparing the articles that gave rise to the identification of each of the terms. For many of these terms, there was practically total overlap between the groups of articles that gave rise to the terms identified as synonyms.

#### Neutral topics

The terms grouped in *neutral topics* were disregarded from the content analysis because they practically returned all the records used in the term detection approach. This result is consistent with what was expected for the technique that uses the formation of matrices to search for terms and, for this reason, unspecific terms have a larger data set.

#### Economic aspects

This topic refers mainly that the macro aspects of the market such as the mostly *a la carte* standard of treatments [29] and a structure with many clinics operating on a small/medium scale of the market that is rivaling the recent and growing formation of conglomerates [30].

Economic aspects are hardly quantifiable in imperfect markets such as health, where there are high interference levels from variables and market regulations [10]. The high prices are only one of several factors determining the ART market, which has cultural and religious associations that cannot be easily measured or evaluated by econometry [31]. However, the data collected on the growth of the ART market size in the last decades indicates that the regional discrepancies are derived from the different attractiveness for the several capital contributions made by different public and private subjects, a phenomenon known as the ’Matthew effect’ [32].

About the stock exchange and merging & acquisitions in the ART market (MAART), a few large companies have spent millions of dollars consolidating a fragmented IVF market [33, 34]. While the conglomerates are growing, more venture capital firms invest in startups and fertility clinics, including specific niches [30]. These expansions reach state and national borders with a more entrepreneurial and corporate bias and heavy investments in technology [35].

Among the main actions carried out by companies in the sector we can mention:

- 2013—An Australian IVF company became the first IVF company traded on a major stock exchange, and it holds about 35% of the market [36, 37].
- 2016—Cooper Surgical acquired Wallace Pharmaceuticals (India) for approximately USD 168 million [38].
- 2017—PitchBook accounted for more than US$ 178 million invested in startups that develop fertility products [34].
- 2017—The merger of IVI-RMA made this company the largest assisted reproduction center worldwide [39].
- 2017—The Thomson Medical Group Ltd. (TMG) formalized a joint venture to expand the IVI-RMA network in APAC and Mexico markets [40].
- 2019 –An enterprise that manages fertility benefits for employees of large companies reached USD 103.4 million in the first semester and released the shares on NASDAQ [41].

Coverage has a significant effect on use for older and more educated women, more significant than the effects found for other groups [42, 43]. Studies report that more than half of working women consider changing jobs for better reproductive health benefits [44].

On the other hand, there is the possibility that insurance coverage laws may have adverse effects on total fertility in the medium and long term due to overly optimistic perceptions about the possibility of extending or delaying reproductive life in an induced way, which can be called ex-ante moral hazard [42, 45], one of the alleged reasons for reducing public funding in Germany and Australia [46, 47].

There is a growth in coverage for infertility treatment among jumbo employers, who tend to be trendsetters for smaller employers, and studies reported that more than half of working women would consider changing jobs for better reproductive health benefits [48, 49]. On the other hand, there is the possibility that policies may have adverse effects on total fertility due to overly optimistic perceptions about the delay of reproductive life [42, 45], one of the alleged reasons for reducing public funding in Germany and Australia [46, 47].

Generally, economic recessions impact natural fertility in the developed world in does not leave a visible mark on the fertility levels of the global cohort [50]. The expressive increase in COVID-19 cases and massive hospitalizations has collapsed most health systems globally and caused the suspension of new fertility treatments, except for patients on cycle or who urgently require fertility preservation for oncological reasons [51].

Although the countries reacted with diverse responses in this pandemic, the ART services have been mainly responsive to public health and individual patient concerns [52]. The pandemic impact on fertility appears to have five main factors: high mortality, restricted access to family planning services, reduced work-life balance, economic recession and uncertainty, and disruptions to assisted reproduction services [53]. It is still early to assess how the pandemic caused by the Covid-19 disease has affected the ART market; however, it is expected that the economic recession and uncertainty impact assisted reproduction services.

#### Compensation for reproductive services

Regarding the topic of c*ompensation for reproductive services*, the separation of these topics, although practical, has limitations because all of them are also strongly related to social aspects and legislation. We found two main analyses in the returned records for this topic: a) gamete pricing, and b) commercial surrogacy. Both are part of a more focused analysis on transnational markets called cross-border reproductive care (CBRC), popularly called reproductive tourism.

About 10% of IVF cycles are performed in the USA with donor eggs [54], and the results are like the use of fresh and frozen oocytes [55]. The term "donation" of gametes is considered inappropriate because they are generally sold [56]. The United Kingdom limits gamete’s values, while gamete donations are banned in Japan [54]. A complex set of stereotypes has led to the monetization of gametes and embryos and rapid response to price stratification based on donor phenotype and social characteristics as a degree or artistic achievements [57–59].

The CBRC is a global billionaire industry phenomenon that involves the transnational *laissez-faire* regulation [60–62], inequalities [63], and the demand for reproductive services [38, 64]. It is a contentious and largely unregulated area [65] governed by the heterogeneity of conditions in each country [66–68]. At least ten motivations for CBRC have already been identified, grouped into four broad categories: legal and religious prohibitions, resource considerations; quality and safety concerns; and personal preferences [69].

#### Geographic distribution

The topic of g*eographic distribution* comprises 9.5% of the citations (12 terms, 739 citations), the most discussed subject in the market reports. In general, the data presented addresses:

- The size of the market in billions of dollars: globally was around USD 26 billion in 2019 [12] and is expected to reach USD 45 billion by 2025 [5];
- Percentage distribution of clinics and number of procedures worldwide: Europe and North America represent ∼65% of the global ART market, followed by APAC with ∼25%; Middle East, Africa, and Latin America (also called by ’rest of the world’—RoW) representing ∼10% [5];
- Procedures and clinics per region: between 1997 and 2016, ART treatments have increased more than five-fold in Europe, 4.6-fold in North America, three-fold in Australia and New Zealand [13, 14], with grown expectative in all scenarios and
- Factors (social/legal/economic) that impact this distribution: increasing infertility rates [7, 15, 16, 38, 70, 71], rising disposable incomes [5, 70–73], adoption of the western lifestyle [16, 73–75], late family planning [16, 70, 72], low-cost and high-quality healthcare [18, 72, 75], favorable government initiatives [7, 38, 76], expansion of healthcare infrastructure [64, 74], reduced socio-ethical stigma [15, 77], and the CBRC [18, 38, 71, 72, 78, 79].

#### ART market projections

After neutral terms, the topic of *ART market projections* focused on more generic terms (Table 1). The content mainly presents forecasts of the contents present in other topics such as social aspects, geographic distribution, accessibility, and regulation. The clustered terms comprised various database content, with analytical content as a characteristic in common. In addition to the market’s financial growth expectations, there is also an assessment of the geographic distribution, with the unanimous affirmation that the Asia-Pacific (APAC) market has the greatest compound annual growth rate (CAGR) and potential [5, 7, 15, 16, 38, 70–75, 77, 78] (S3 Table).

The stock exchanges participation and mergers & acquisitions in the ART market (MAART) are a trend observed for a few large companies that have spent millions of dollars to consolidate a fragmented IVF market [33, 34], with heavy investments in technology [35]. These companies also have been focused on specific niches considered non-traditional families [30].

The main projected concerns relate to reproductive commodification, in particular commercial surrogacy, and stereotypic gamete pricing [63, 68, 80–82]. In the same way that India regulated the issue to protect vulnerable women groups [65, 83], there is a debate about ways of fair compensation for domestic surrogacy in Australia, the introduction of professional intermediaries, and limits on advertising to minimize risks [84]. It is an issue that is difficult to resolve and that depends on efforts and intranational agreements.

#### Social aspects

The topic s*ocial aspects* subgroups can be clustered into five subgroups: a) ethical/moral discussions (4 terms, 314 citations); b) religious issues (3 terms, 84 citations); c) gender issues (3 terms, 82 citations); d) and sexual preferences (2 terms, 36 citations); and e) stress was considered an independent term (10 citations).

The records returned in this topic showed considerable overlap with the records returned in the accessibility topic, which is understandable because inequities are strongly associated with social and cultural characteristics [85, 86].

It is complex to measure these social aspects’ impact on the ART market, a complex and imperfect health business where there are high interference levels [10]. The high prices are only one of several factors determining the ART market, which has cultural and religious associations that cannot be easily measured or evaluated by econometry [31].

It is estimated that a 1% increase in European national gross domestic product (GDP) would be able to increase 382 ART cycles per million women of reproductive age and, even so, it only increases 25% of this potential, concluding it is due to the social factors involved [87]. These factors also affect nations’ repayment policy (pro-natal or anti-natal) regardless of their GDPs, reflecting cultural and social priorities [88].

Many records about queer reproductive justice (QRJ) are returned on this topic. It refers to non-normative audiences who want to form a family nucleus, such as homo-affective couples, single parents, and other audiences who are discouraged when seeking reproductive services [89, 90]. This market niche is often not directly related to accessibility and cost problems, and its acceptance has been partly driven globally by the strength of the neoliberal market [91, 92].

#### Regulation

Despite representing 6.7% of the number of citations in the detection stage, the topic of *regulation* represented 13% of the corresponding bibliography. Around 85 of those contributing to the IFFS triennial publication have regulated legislation or guidelines [65], generally associated with national economic and moral concerns [93].

**Fig 4:**
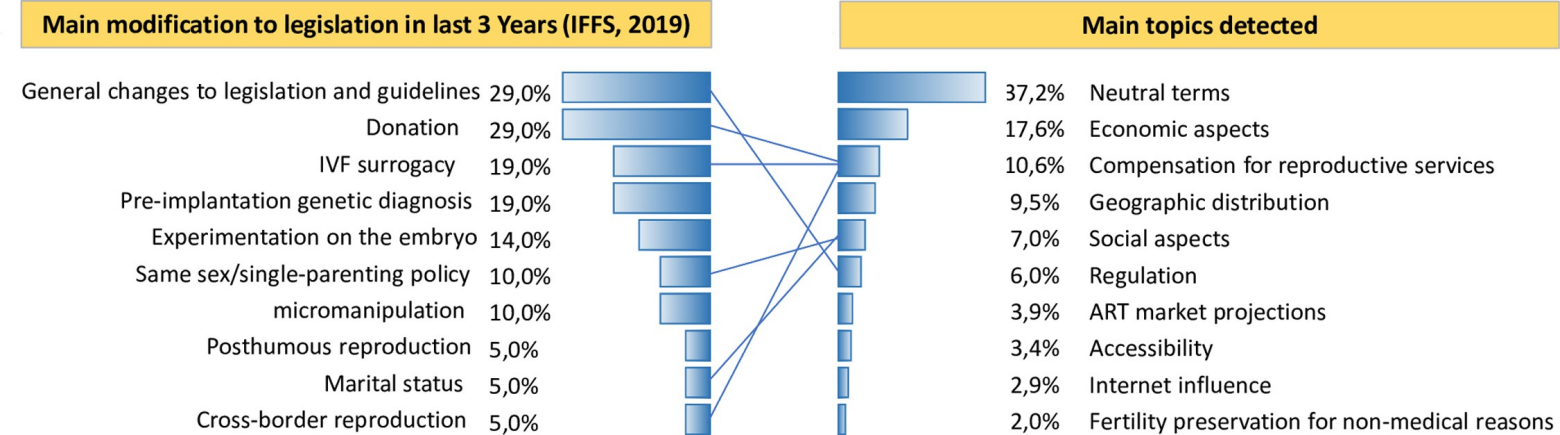
Location of the main themes of legislative changes within the main topics identified. The main themes of recent legislative changes stated by the IFFS (2019) coincide with the terms identified by topic modeling. In this way, we identified in which topics the similar terms were inserted and prepared this graph to illustrate the location of the main themes of legislative changes in relation to the main topics in assisted reproduction. Note that each topic may be included in more than one topic due to the complexity of the market.

We compared the data presented on the main modification to legislation in the last three years with the main topics presented by IFFS [65] detected, to assess whether there is synchronism in the topics assessed in legislation and academics (Fig 4). We found some common points, but no direct correlation in the timing of the discussions.

There is a recent debate on global policy and systematic regulatory forecasts to guide government responses to the existing market, preferably including a discussion open to all interested parties [94, 95]. Most of the articles cited, regardless of the central focus, conclude with the statement of the need for consensual regulation at a global level to regulate the market and the public, thus avoiding most ethical conflicts [61, 63, 68, 96, 97].

Based on this lack of regulation, incoherent or fragmented regulation, the ART market worldwide is provided by free-market initiatives [63] and is associated with themes such as CBRC [98] and embryo gender selection [79, 99]. Although it is a consensus that current regulations do not guarantee the exercise of reproductive rights and equal opportunities [100, 101].

#### Accessibility

The topic of *accessibility* has the same number of terms as the *regulation* topic but almost half the number of citations (9 terms, 264 citations). It is a topic strongly associated with economic aspects and regulation, as noted in the definition itself, which is the level of access to medical treatments necessary for infertility care through vantage/disadvantage in other aspects such as financial/social, race, class, gender, culture, and legal status played out on a social field [67, 86].

Two terms that compile the dynamic and synergistic balance in the ART market, both domestic and transnational, are reproductive governance and stratified reproduction. Reproductive governance defines how different social actors use their powers to produce reproductive behaviors, such as legislative controls or permissions, economic incentives or disincentives, ethical and moral injunctions, or inductions [102]. Stratified reproduction refers to the inequity in reproductive rights by race, class, gender, culture, and legal status played out on a social field [67] that generates the accessibility and treatment offered to people into separate groups [85, 86].

Accessibility, the ratio of the cost of IVF treatment to annual income [88], affects not only who can have access to ART treatment but also a) which treatments are used, as cheaper techniques are generally more likely to be covered by health insurance 100, and b) how ART is practiced, such as the association between accessibility and the number of embryos transferred [46]. This cascade of decisions impacts the results [10], and, still, most patients bear partial costs [103].

In IFFS 2019 Vigilance, 62% of the countries reported no existing family concept ART requirements; however, 50% reported limiting access to diagnostic or treatments mainly to single women or same-sex couples, excluding single men and intersex or transgender subjects [65]. This market niche is often not directly related to cost, and its acceptance has been partly driven globally by the strength of the neoliberal market [91, 92].

#### Internet influence

The topics of *Internet influence* and *fertility preservation for non-medical reasons* or *social egg freezing* (SEF) comprise less than 3% of the citations each. However, both are frequently cited in the texts included in the topic *trends & concerns*, where both growing trends and sources of concern and attention are pointed out. It is common sense that the internet and social media are powerful tools of massive influence, used by most patients during their infertility journey [104, 105]. The content of these sites influences consumers’ selection process of both the chosen clinic and the doubts and desires for treatment and the possibility of high expectations [106]. The Society for Assisted Reproductive Technology (SART) updated your policy in 2018 to reduce public misunderstandings caused by different interpretations of data provided by clinics [105].

In addition to the search for information, the internet and social media have become spaces for selling surrogacy services in countries with legal permission or omission. This happens through forums for possible substitutes and customers [63] and a rapidly growing market for SEF and human commodities. While the benefit of dissemination and information is clear, it is essential to ensure that there is no misrepresentation and distribution of misleading information [107].

#### Fertility preservation for non-medical reasons

Despite being the least represented among the topics (5 terms, 156 citations), s*ocial freezing* is one of the main trends [108]. Some jumbo enterprises announced the social freezing as a workplace benefit [48, 49], although the American Society for Reproductive Medicine (ASRM) guideline includes a caution to avoid false hopes about delaying procreation [104]. The main reasons are not having a committed [109, 110]^,^ searching for financial security via career, or completing studies [111, 112].

The possibility of preserving fertility in healthy women as a precaution for future infertility has gained strength in recent years [108] and the case of reproductive preservation in trans individuals who intend to alter their hormonal system and reproductive organs [113].

The emergence of egg banking can be considered a different sector in the infertility industry [114]. The influence of media and the desire for women’s autonomy contributed to the market growth [5, 48, 49, 114–116]; this focuses on the public after 30, a suboptimal age from a clinical point of view, because the quantity and quality of eggs have already decreased considerably [108].

The ASRM guideline on ART marketing includes a caution to avoid false hopes about delaying procreation, which falls short of what is requested regarding the type and quality of information on most affiliated clinic sites [48, 104, 117].

In parallel, in several situations, the comparative analysis of cost-effectiveness based on direct medical costs demonstrates that the SEF can be financially advantageous in comparison to IVF in older women [118, 119]. However, the most efficient/economic strategy for women planning to postpone pregnancy remains uncertain [117].

## Conclusions

The division into clusters was helpful for the identification of topics and do not limit the evaluation of the behavior of the global market, as is the case of the notorious association between moral concerns and national legislation [121]. Topic modeling proved to be an appropriate tool for detecting terms that allowed us to cluster relevant aspects of this growing market. We were able to identify the size and distribution of this market, as well as list legal, social, and economic aspects, as well as trends and concerns.

Analyzing the ART market is a challengesince many isolated, interdependent, and feedback factors compose it, with cultural and religious associations that cannot be easily evaluated by econometry [31]. We note that most studies conclude on the need for transnational regulations to solve different issues. We also highlight, the need of more actions in terms of Corporate Social Responsibility, in which the commitment of companies to society occurs based on the practices carried out, going beyond the concept of profits [120].

We found that, most of the works addresses economic, regulatory, and geographic aspects, and that these topics covered have a synergistic relationship with each other. Two findings gained special attention: a) the potential impact of the formation of conglomerates and mergers on a transnational scale (MAART), considering the certainty about the growing search for reproductive treatments even with legal/social/financial barriers for the final consumer, this has a potential impact on the fragmented pattern of small/medium scale operation, as well as on the CBRC; and b) the lack of health technology assessment (HTA) in reproductive add-ons. Despite technological advances and the insertion of many add-ons over two decades, the success rate remains at around 30% of IVF cycles [14], especially considering that the ART market devices & consumables were valued at USD 13.75 Billion in 2020 and projected to double by 2028 [121].

From these findings, it will be possible to establish dynamic and synergistic relationships between the identified topics. This can be used to generate predictive models about the ART market and to point out situations that need to be better understood, such as the low efficiency of IVF cycles. This information can help identify new market niches and increase the availability of technologies and actions for the treatment of infertility.

## Limitations of the study

The most significant limitation of this study is the impossibility of exhausting each identified aspect. Also, the generalization of accumulated data causes the loss of local nuances. We would like to create correlation cascades, but we chose not to do so at the risk of creating spurious contexts and escaping the intended purpose of the scoping review.

## Supporting information

S1 Table Preferred Reporting Items for Systematic reviews and Meta-Analyses extension for Scoping Reviews (PRISMA-ScR) Checklist. The elaboration of reviews from the checklist is mandatory for quality studies. We used the specific model for scope reviews according to the model published by Tricco *et al*. (2018).
(DOCX)

S2 Table Topics detected by automation tool. Complete list of terms mined by topic modeling (LDA protocol by Knime). We identified 121 terms covering 7,806 citations.
(DOCX)

S3 Table The CAGR of the global ART market, according to market reports. Survey of compound annual growth rate (CAGR) presented in market reports on the subject studied. Note the market growth forecast in all scenarios.
(DOCX)
