# Supplementary material for: Main topics in assisted reproductive market: A scoping review
Source: PLoS One. 2023 Aug 1;18(8):e0284099. doi: 10.1371/journal.pone.0284099 (PMC10393141; doi:10.1371/journal.pone.0284099)
Supplement: S2 Table — Complete list of terms mined by topic modeling (LDA protocol by Knime). We identified 121 terms covering 7,806 citations. (DOCX) [file pone.0284099.s002.docx]

| S2 Table. Topics detected. | |
| --- | --- |
| **TOPIC EXTRACTED** | **COUNT** |
| reproductive | 2101 |
| industry | 586 |
| surrogacy | 345 |
| fertilization | 240 |
| donation | 201 |
| globally | 271 |
| services | 172 |
| tourism | 136 |
| ethical | 133 |
| access | 128 |
| marketing | 117 |
| private | 107 |
| social | 99 |
| India | 98 |
| regulation | 98 |
| markets | 97 |
| commercial | 91 |
| cross-border | 85 |
| increase | 83 |
| countries | 74 |
| economy | 71 |
| europe | 70 |
| report | 67 |
| freezing | 66 |
| legal | 66 |
| society | 66 |
| public | 62 |
| catholic | 60 |
| websites | 60 |
| insurance | 58 |
| perspectives | 52 |
| transnational | 52 |
| issues | 49 |
| travel | 48 |
| implications | 47 |
| policy | 47 |
| gender | 46 |
| media | 43 |
| effects | 41 |
| coverage | 40 |
| influence | 39 |
| china | 35 |
| employment | 32 |
| contraceptive | 30 |
| internet | 30 |
| justice | 30 |
| decision-making | 28 |
| gestational | 28 |
| preservation | 28 |
| inequalities | 26 |
| queer | 26 |
| costs | 25 |
| federal | 25 |
| conceive | 24 |
| unregulated | 24 |
| patient | 23 |
| genetic | 22 |
| parental | 22 |
| guidelines | 21 |
| quality | 21 |
| banking | 20 |
| concerns | 20 |
| exploitation | 20 |
| management | 20 |
| perceptions | 20 |
| reduced | 20 |
| spain | 20 |
| providers | 19 |
| conception | 18 |
| diversity | 18 |
| experience | 18 |
| lesbian | 18 |
| online | 18 |
| wechat | 18 |
| worldwide | 18 |
| opportunities | 17 |
| abortion | 16 |
| carrier | 16 |
| choice | 16 |
| clock | 16 |
| constraints | 16 |
| decline | 16 |
| demand | 16 |
| improvements | 16 |
| information | 16 |
| israel | 16 |
| middle | 16 |
| moral | 16 |
| official | 16 |
| population | 16 |
| trends | 16 |
| adoption | 15 |
| delivery | 15 |
| acceptability | 14 |
| considerations | 14 |
| created | 14 |
| equity-affiliated | 14 |
| payment | 14 |
| potential | 14 |
| religion | 14 |
| benefits | 13 |
| comparison | 13 |
| stratified | 13 |
| business | 12 |
| commodification | 12 |
| disparities | 12 |
| latin | 12 |
| recession | 12 |
| conditions | 11 |
| concern | 10 |
| counselling | 10 |
| estimated | 10 |
| feminist | 10 |
| financial | 10 |
| methods | 10 |
| pregnant | 10 |
| religious | 10 |
| stress | 10 |
| banks | 8 |
| controls | 8 |
| facilitators | 8 |

Complete list of terms mined by topic modeling (LDA protocol by Knime). We identified 121 terms covering 7,806 citations.
