# Supplementary material for: Main topics in assisted reproductive market: A scoping review
Source: PLoS One. 2023 Aug 1;18(8):e0284099. doi: 10.1371/journal.pone.0284099 (PMC10393141; doi:10.1371/journal.pone.0284099)
Supplement: S3 Table — Survey of compound annual growth rate (CAGR) presented in market reports on the subject studied. Note the market growth forecast in all scenarios. (DOCX) [file pone.0284099.s003.docx]

| S3 Table. The CAGR of the global ART market, according to market reports. | | | |
| --- | --- | --- | --- |
| **MARKET REPORT** | **START DATE** | **END DATE** | **CAGR (%)** |
| (Grand View Research, 2020) | 2020 | 2027 | 4.7 |
| (Global Market Insights *et al.*, 2020) | 2020 | 2026 | 4.4 |
| (Market Research Future, 2020) | 2020 | 2023 | 10.0 |
| (Allied Market Research, Sumant, *et al.*, 2019) | 2020 | 2027 | 15.9 |
| (Allied Market Research, Shaikh, *et al.*, 2019) | 2019 | 2026 | 16.6 |
| (Report Buyer, 2019) | 2019 | 2027 | 6.39 |
| (Verified Market Research, 2019) | 2019 | 2026 | 6.27 |
| (Grand View Research, 2018) | 2018 | 2025 | 10.0 |
| (Markets and Markets, 2018) | 2018 | 2023 | 8.0 |
| (Mordor Intelligence, 2017) | 2018 | 2025 | 2.0 ^*technologies^ |
| (Markets and Markets, 2017a) | 2017 | 2022 | 10.0 |
| (Markets and Markets, 2017b) | 2016 | 2022 | 10.9 |

Survey of compound annual growth rate (CAGR) presented in market reports on the subject studied. Note the market growth forecast in all scenarios.
